# Supplementary material for: The Systems Biology Research Tool: evolvable open-source software
Source: BMC Syst Biol. 2008 Jun 29;2:55. doi: 10.1186/1752-0509-2-55 (PMC2446383; doi:10.1186/1752-0509-2-55)
Supplement: Additional file 1 — SBRT Archive. An archive of the current version of the Systems Biology Research Tool. [file 1752-0509-2-55-S1.zip › sbrt-1.4.0/doc/developers_guide/api/sbrt/shell/mng/fba/ExtremeCurrentManager.Rm.html]

ExtremeCurrentManager.Rm


|  |  |  |
| --- | --- | --- |
| **Overview** \| **Package** \| **Class** \| **Use** \| **Tree** \| **Deprecated** \| **Index** \| **Help** | |  |
| **PREV CLASS**   **NEXT CLASS** | **FRAMES**    **NO FRAMES**     **All Classes** |  |
| SUMMARY: NESTED \| FIELD \| CONSTR \| METHOD | DETAIL: FIELD \| CONSTR \| METHOD |  |


---


## sbrt.shell.mng.fba Class ExtremeCurrentManager.Rm

```
java.lang.Object
  sbrt.kernel.io.AbstractResultsManager<DoubleMatrix<?,java.lang.String>>
      sbrt.shell.mng.fba.ExtremeCurrentManager.Rm
```

**All Implemented Interfaces:**: ResultsManager<DoubleMatrix<?,java.lang.String>>

**Enclosing class:**: ExtremeCurrentManager

---

``` protected static class ExtremeCurrentManager.Rm extends AbstractResultsManager<DoubleMatrix<?,java.lang.String>> ```

**Author:**
:   wright

---

| **Constructor Summary** | |
| --- | --- |
| `protected` | `ExtremeCurrentManager.Rm(ComoFile<java.lang.String,java.lang.Double> output)` |


| **Method Summary** | |
| --- | --- |
| `void` | `addData(DoubleMatrix<?,java.lang.String> results)`             Adds data to this results manager. |

**Methods inherited from class sbrt.kernel.io.AbstractResultsManager**

`breakPoint, checkState, close, getName, isClosed`

**Methods inherited from class java.lang.Object**

`clone, equals, finalize, getClass, hashCode, notify, notifyAll, toString, wait, wait, wait`

**Methods inherited from interface sbrt.kernel.io.ResultsManager**

`addData`

**Constructor Detail**

### ExtremeCurrentManager.Rm

```
protected ExtremeCurrentManager.Rm(ComoFile<java.lang.String,java.lang.Double> output)
```

**Parameters:**: `output` -


**Method Detail**

### addData

```
public void addData(DoubleMatrix<?,java.lang.String> results)
```

:   Adds data to this results manager.

    :   **Parameters:**: `results` - the results, or data, to be added to this manager. **Throws:**: `java.lang.IllegalStateException` - if `close()` has already been called.


---


|  |  |  |
| --- | --- | --- |
| **Overview** \| **Package** \| **Class** \| **Use** \| **Tree** \| **Deprecated** \| **Index** \| **Help** | |  |
| **PREV CLASS**   **NEXT CLASS** | **FRAMES**    **NO FRAMES**     **All Classes** |  |
| SUMMARY: NESTED \| FIELD \| CONSTR \| METHOD | DETAIL: FIELD \| CONSTR \| METHOD |  |


---
